# Prediction of histone deacetylase inhibition by triazole compounds based on artificial intelligence

**DOI:** 10.3389/fphar.2023.1260349

**Published:** 2023-11-15

**Authors:** Yiran Wang, Peijian Zhang

**Affiliations:** College of Computer Science and Technology, Qingdao University, Qingdao, Shandong Province, China

**Keywords:** cancer, HDAC inhibition, quantitative structure-activity relationship, support vector machine, particle swarm optimization

## Abstract

A quantitative structure-activity relationship (QSAR) study was conducted to predict the anti-colon cancer and HDAC inhibition of triazole-containing compounds. Four descriptors were selected from 579 descriptors which have the most obvious effect on the inhibition of histone deacetylase (HDAC). Four QSAR models were constructed using heuristic algorithm (HM), random forest (RF), radial basis kernel function support vector machine (RBF-SVM) and support vector machine optimized by particle swarm optimization (PSO-SVM). Furthermore, the robustness of four QSAR models were verified by K-fold cross-validation method, which was described by *Q* ^*2*^. In addition, the *R* ^*2*^ of the four models are greater than 0.8, which indicates that the four descriptors selected are reasonable. Among the four models, model based on PSO-SVM method has the best prediction ability and robustness with *R* ^*2*^ of 0.954, root mean squared error (RMSE) of 0.019 and *Q* ^*2*^ of 0.916 for the training set and *R* ^*2*^ of 0.965, RMSE of 0.017 and *Q* ^*2*^ of 0.907 for the test set. In this study, four key descriptors were discovered, which will help to screen effective new anti-colon cancer drugs in the future.

## 1 Introduction

Colon and rectal cancers are the most common gastrointestinal tumors (Chen et al., 2019; Pan et al., 2023). Currently, colon cancer is one of the most common solid malignancies, which is the third leading cause of cancer-related new cases and deaths worldwide (Chen et al., 2019; Shi et al., 2022a; Shi et al., 2022b). Most patients with colon cancer present with advanced disease, whose survival rate is very low. More than 95% of the patients with colorectal cancer in the diagnosis of aged 50 or older, and the overall survival rate for advanced, metastatic, and recurrent colon cancer is less than 50%. (Wang et al., 2015; Shi et al., 2022a). Due to the numerous factors in the development and progression of colon cancer, its pathogenesis is still unclear. Current treatment options for colon cancer include surgery, chemotherapy, and molecularly targeted therapy. Treatments for colon cancer are limited, and a large percentage of patients develop resistance to current treatments (Schmoll and Stein, 2014). Therefore, the study of new therapeutic strategies and the development of new drugs are the key points in the research of colon cancer (Xiong et al., 2023).

At present, an emerging therapeutic approach for colon cancer is the use of corresponding histone deacetylase (HDAC) inhibitors (Tavares et al., 2017). HDAC is a kind of epigenetic antitumor drug targets (Choi et al., 2023). Because of the important role of HDAC in various biological processes such as cell proliferation, metastasis and apoptosis, HDAC inhibitors has been widely studied as a novel anticancer drug target (Gillette, 2021; Roy et al., 2023). Though HDAC inhibitors have not been approved by FDA to treat colon cancer, some preclinical studies have discovered its efficacy to treat colon cancer *in vitro* and *in vivo* (Kang et al., 2009; Asklund et al., 2012; Yao et al., 2014). However, there are some limitations of current approved HDAC inhibitors, such as pan-inhibition, etc. Thus, it is in urgent need to develop novel HDAC inhibitors to improve colon cancer treatment (Place et al., 2005; Sang et al., 2023) Nan Sun et al. designed and synthesized a series of triazole-containing compounds as novel HDAC inhibitors, which have significant anti-proliferation effect on murine and human colon cancer cell lines MC38 and HCT116 (Sun et al., 2023). In the studies of discovering new HDAC inhibitors, the measurement of HDAC inhibition IC_50_ values of compounds has great influence for the design of new effective anti-colon cancer drugs. Since numerous chemical experiments are costly and time-consuming, a new and effective method for predicting the IC_50_ of untested compounds should be found (Zhao et al., 2020).

In 1964, the concept of the quantitative structure-activity relationship (QSAR) was first proposed by Free et al. and then was widely used (Free and Wilson, 1964; Hansch and Steward, 1964; Myint and Xie, 2010). QSAR is based on the general principle of medicinal chemistry that the biological activities of a ligand or compound is related to its molecular structure or properties, and molecules with similar structures may have similar biological activities (Myint and Xie, 2010). Model established based on QSAR can reveal quantitative structure-activity relationship between biological activities and part of descriptors of set of known compounds with similar structure (Huang et al., 2021). Then QSAR model can be used to predict the activities of unknown compounds that have similar structure with the previous known compounds, which has widely used in the process of screening out efficient and novel drugs (Sun et al., 2023).

Therefore, four QSAR models were established in this study to predict the HDAC inhibition IC_50_ of 60 selected compounds based on descriptors selected by the heuristic method (HM). The methods establishing models in study are HM, random forest (RF), support vector machine with radial basis kernel function (RBF-SVM) and RBF kernel function support vector machine with particle swarm optimization (PSO-SVM). In addition, the HM method was also used to select descriptors. Among the four models, the model constructed by PSO-SVM has the best performance and strongest robustness. In addition, the *R* ^*2*^ of the established four models meet the requirement of predicting IC_50_ of compounds, which indicates the descriptors used in the models were enough and good for drug design. Overall, this study will provide efficient guidance and help for the screening of a new type of colon cancer drug.

## 2 Material and methods

### 2.1 Data set

The 60 compounds containing triazole studied in this paper are all from the same literature, which eliminates unforeseen problems due to data from different sources^17]^. The IC_50_ value of HDAC inhibition was determined by Sun et al. after exposing the compound to the same experimental environment for 72 h to ensure the accuracy of the experiment^17^. The compounds used in this study and their IC_50_ values are shown in Supplementary Table S1. All compounds were randomly divided into the training set and test set in the ratio of 4:1, of which 48 compounds in training set were used to construct models and the remaining 12 compounds in test set were used to evaluate the performance of the models (Fan et al., 2018).

### 2.2 Calculating descriptors

The calculation of molecular descriptors and the selection of appropriate molecular descriptors are the key prerequisites of establishing QSAR models, which directly affects the performance of QSAR models, such as accuracy of prediction. Comprehensive descriptors for structural and statistical analysis (CODESSA) is currently the more common package to calculate molecular descriptors and perform statistical analysis (Katritzky et al., 2005; Zhang et al., 2013). The steps for calculating and selecting molecular descriptors are as follows. Firstly, ChemDraw was used to draw the structure of the compounds to get mol file and skc file (Evans, 2014). Secondly, the compounds in mol format were optimized using HyperChem software under the guidance of the theory that the lower the energy of a molecule is, the more stable its structure is. In the optimization process, MM+ molecular mechanical force field was used to preliminarily optimize the compound, and then semi-empirical AM1 method was used to further optimize the compound to obtain the most stable structure, which is beneficial to improve the calculation accuracy of molecular descriptors (Lima et al.). The optimized structures by HyperChem were stored in hin and zmt formats as the input of MOPAC. After that, MOPAC was used to generate mno files as the input files of CODESSA to calculate descriptors. Five categories of molecular descriptors were obtained using CODESSA, which are structural, topological, geometric, electrostatic, and quantum chemical (Coi et al., 2006; Madugula and Yarasi, 2017).

### 2.3 Linear model by HM

HM is a common and effective method for selecting descriptors which was widely used at present (Wang et al., 2020; Gao et al., 2022). This method is not limited by the size of data set, and is highly efficient (Yang et al., 2023; Li et al., 2023). Therefore, HM was used to select the appropriate descriptor from the specific descriptors computed by CODESSA. This method can select the descriptors responsible for activity from the descriptor set by building a multiple linear regression models. The following descriptors should be excluded before building linear regression models by HM. 1) Special descriptors that not all compounds possess. 2) Descriptors with correlation coefficients greater than 0.8, namely collinear descriptors.

In this study, square of correlation coefficient (*R* ^*2*^) and root mean square error (RMSE) were used to evaluate and analyze the performance of models established by HM, RF, SVM and PSO-SVM. In addition, the robustness of the models were verified by K-fold cross-validation.

### 2.4 Nonlinear model by RF

The IC_50_ value of HDAC inhibition is influenced by many factors, so the linear model cannot accurately predict the IC_50_ of HDAC. Therefore, three kinds of nonlinear models were established by RF, SVM and PSO-SVM. Random forest is a supervised machine learning algorithm based on ensemble learning (Feng et al., 2019). It can effectively reduce the risk of overfitting and is more conducive to obtain a robust model. Therefore, it is a novel and efficient method to establish QSAR nonlinear models (Zhang et al., 2015a; Fang et al., 2022.).

The steps to build a RF regression model are: 1) First, build a dataset containing the descriptor values and -lg (IC_50_) values for training set and test set. 2) Build RF model. Set the number of decision trees to control the RF’s behavior (Li et al., 2012; Song, 2015; Ao et al., 2019). 3) Train the RF using the training set. RF method constructed multiple decision trees based on descriptor data and IC_50_ values in training set, and performs feature selection and partitioning in each tree. 4) Use the trained RF model to predict the samples in the test set. In this model, the prediction results of each decision tree were weighted to obtain the predicted IC_50_ value. 5) Use a variety of performance indicators to evaluate prediction accuracy and generalization ability of RF model.

### 2.5 Nonlinear model by RBF-SVM

Support vector machine (SVM) proposed by Vapnik and colleagues in 1964 is a generalized linear classifier that classifies data through supervised learning (Girosi, 1998). SVM can also be used for regression, which is called support vector regression (SVR) (Santamaria-Bonfil et al., 2016). The main idea of SVR can be summarized as transforming the linearly inseparable samples of the low-dimensional input space into the high-dimensional feature space by using the nonlinear mapping algorithm, so that the linear regression can be performed in the high-dimensional feature space (Collobert and Bengio, 2001; Khemchandani and Jayadeva, 2009; Huang and Zhao, 2018).

By introducing ε-insensitive loss function, regularization constant *C*, relaxation variable $\xi_{i}$ , ${\hat{\xi }}_{i}$ , normal vector *w*, displacement *b* to be determined, the SVR is formalized as follows in Eq. 1, and the constraint as follows in Eq. 2:

$$f\left(x\right)=\min_{w,b,\xi_{i},\hat{\xi }}\frac{1}{2}{\left\|w\right\|}^{2}+C\sum_{i=1}^{m}\left(\xi_{i}+{\hat{\xi }}_{i}\right)$$ (1)

$$s.t.\left\{\begin{array}{l} w^{T}\cdot x_{i}+b-y_{i}\leq \varepsilon +\xi_{i},\\ y_{i}-w^{T}\cdot x_{i}-b\leq \varepsilon +\hat{\xi }\\ \xi_{i}\geq 0,\hat{\xi }\geq 0,i=1,2...m \end{array}\right\}$$ (2)

Therefore, the final linear regression function of SVR is obtained as shown in Eq. 3:

$$f\left(x\right)=\sum_{i=1}^{m}\left({\hat{\alpha }}_{i}-\alpha_{i}\right)\kappa \left(x_{i}^{T}x\right)+b$$ (3)

In Eq. 3, $\kappa \left(x_{i}^{T}x\right)$ is the kernel function, $\alpha_{i}$ and ${\hat{\alpha }}_{i}$ are Lagrange multipliers. Several kernel functions commonly used in support vector machines include linear kernel function, polynomial kernel function, radial basis kernel function and so on. The core idea of RBF is to map each sample point to an infinite dimensional feature space, so as to make linearly indivisible data linearly separable. It is the most commonly used kernel function and shown in Eq. 4:

$$\kappa \left(x_{i},x\right)=\exp\left(-\gamma {\left\|x_{i}-x\right\|}^{2}\right)$$ (4)

where **γ** is a hyperparameter of a radial basis kernel function.

### 2.6 Nonlinear model by PSO-SVM

Because complexity of optimizing many parameters in RBF-SVM is high, particle swarm optimization (PSO) algorithm was introduced in the parameter optimization process, which can converge to the global optimal solution with high efficiency. PSO was proposed by Kennedy and Eberhart in 1995, which is one of the most widely used optimization algorithm (Eberhart and Shi, 2004; Zhang et al., 2015b).

The basic concept of PSO is derived from the study of the foraging behavior of birds (Gong et al., 2016; Bonyadi and Michalewicz, 2017; Pervaiz et al., 2021). PSO can be expressed as: each particle can be regarded as a search individual in the N-dimensional search space, which iterates continuously, updates the speed and position, and finally obtains the optimal solution satisfying the termination condition (Li et al., 2002; Lin et al., 2008; Subasi, 2013). The formula for PSO update speed and location is shown below:

$$V_{id}=\omega V_{id}+C_{1}random\left(0,1\right)\left(P_{id}-X_{id}\right)+C_{2}random\left(0,1\right)\left(P_{gd}-X_{id}\right)$$ (5)

$$X_{id}=X_{id}+V_{id}$$ (6)

where ω is the inertial factor that specifies search step size. *C*_*1*_ and *C*_*2*_ are acceleration constants, *X*_*id*_ represents the d-dimensional position of each particle *i*, and *P*_*gd*_ represents the D-dimension of the global optimal solution.

## 3 Result

### 3.1 Results of HM

**FIGURE 1:**
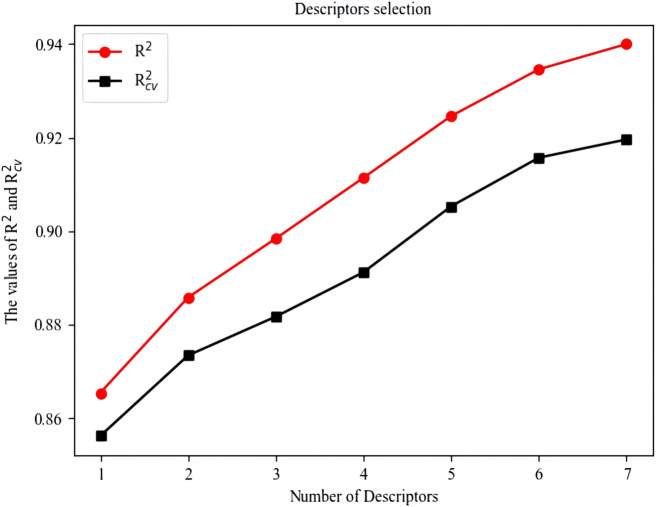
Influence of the Number of descriptors on *R* ^*2*^ and *R*_*cv*_^*2*^.

579 descriptors were calculated by CODESSA. To obtain several descriptors most relevant to the HDAC inhibition, the number of molecular descriptors in linear models were increased from 1 to 7, and the corresponding *R* ^*2*^ and *R*_*cv*_^*2*^ were recorded. Considering that excessive selection of descriptors is not conducive to drug screening and design, the number of descriptors were determined to be 4 of which *R* ^*2*^ and *R*_*cv*_^*2*^ both reached about 0.9. The influence of the number of descriptors on *R* ^*2*^ and *R*_*cv*_^*2*^ is shown in Figure 1.

The four selected molecular descriptors and their physicochemical meanings are shown in Supplementary Table S2. Their correlation coefficients are shown in Supplementary Table S3, all of which are less than 0.8.

The multiple regression linear model established by HM, is shown in Eq. 7.

$$‐\mathrm{lg}\left({\text{IC}}_{50}\right)=‐0.351132‐2.165127*d_{1}+0.940460*d_{2}‐0.669793*d_{3}+0.800716*d_{4}$$ (7)

where *d*_*1*_, *d*_*2*_, *d*_*3*_ and *d*_*4*_ represented MERHN, MREHN, MNRIN and MVO, respectively.

**FIGURE 2:**
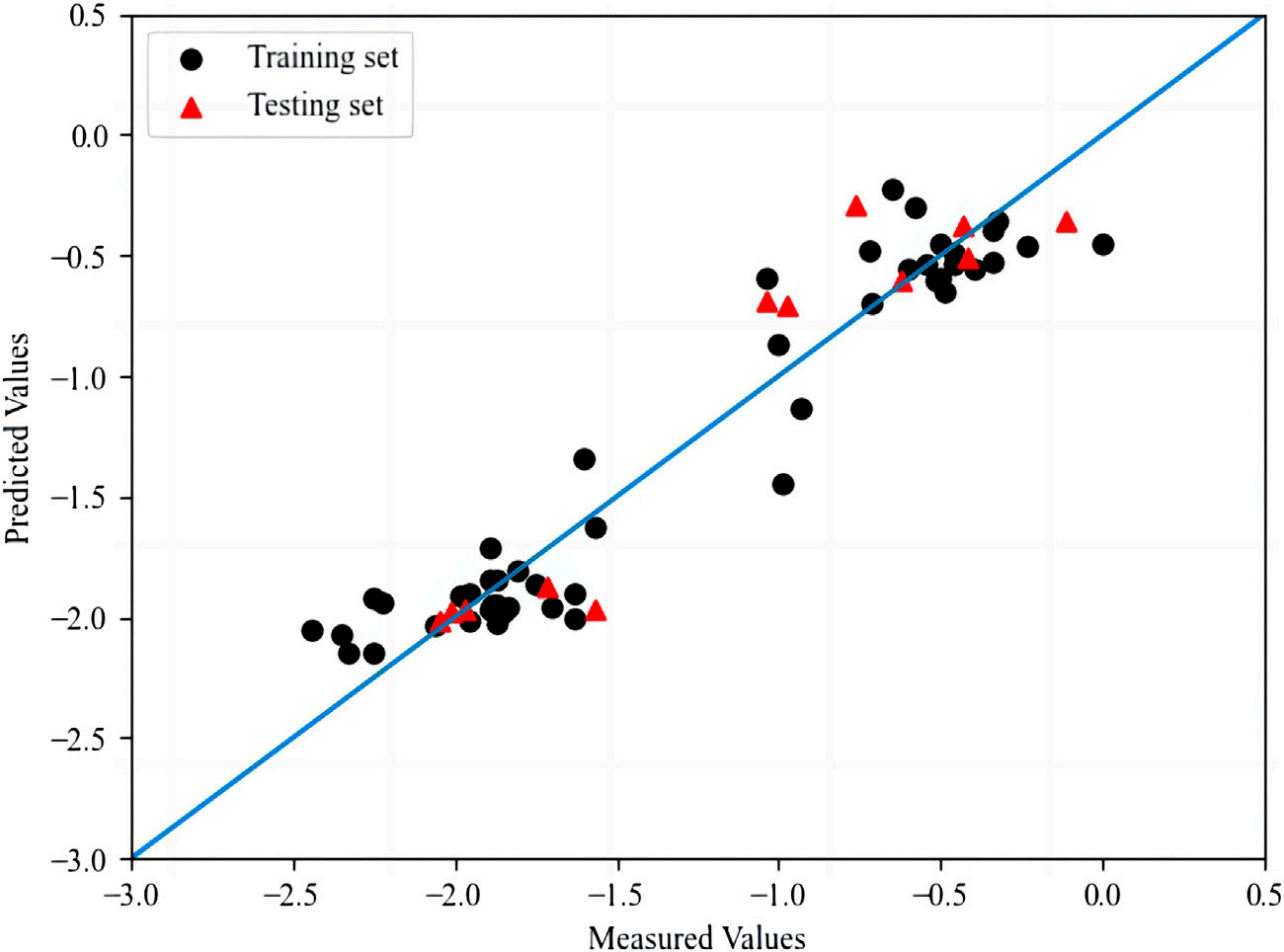
The plot of measured and predicted -lg (IC_50_) by HM.

The *R* ^*2*^ and RMSE of the training set and the test set in the model are 0.917, 0.832 and 0.044, 0.056 respectively. The plot for HM model is shown in Figure 2. In addition, the *Q* ^*2*^ of the training set and the test set are 0.832 and 0.804 respectively through K-fold cross-validation, where the *K* value is 5.

### 3.2 Results of RF

In order to ensure that the results of HM, RF, RBF-SVM and PSO-SVM can be compared with each other, the 4 descriptors selected by HM were used to build other three models by RF, RBF-SVM and PSO-SVM.

When using the RF method, it is necessary to determine the values of some parameters in RF, such as the number of decision trees *n*, the maximum depth of the tree *d*, the number of samples contained in each internal node *s*_*1*_, and the number of samples contained in each leaf node *s*_*2*_. Among them, the larger *n* is, the better the effect of the model tends to be. However, when *n* is larger to a certain extent, the decision boundary is reached, and the accuracy of the random forest usually stops rising or starts to fluctuate.

**FIGURE 3:**
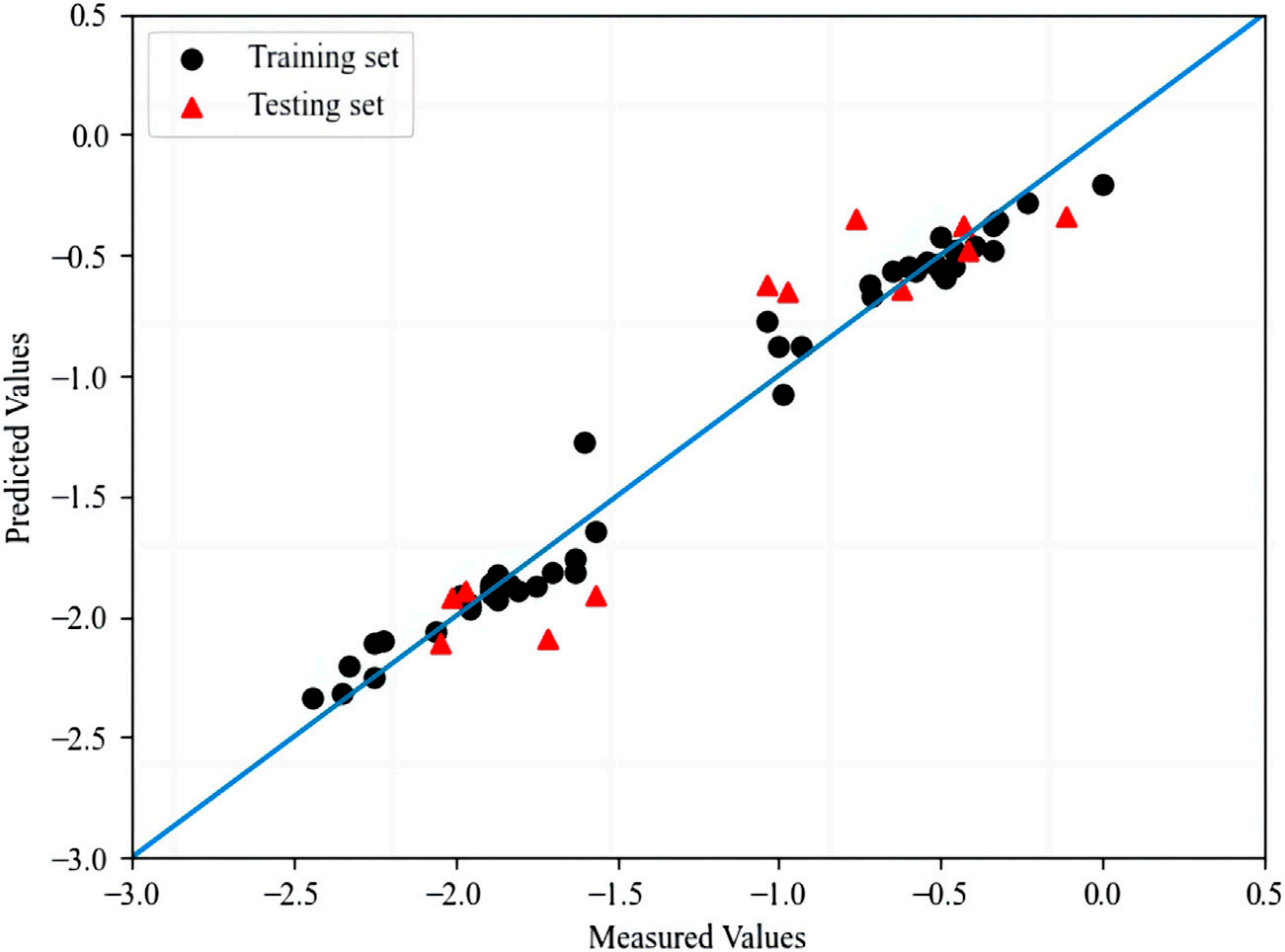
The plot of measured and predicted -lg (IC_50_) by RF.

Set *n* to 100, d to the default value of the python library, *s*_*1*_ to 2, and *s*_*2*_ to 1. The *R* ^*2*^ and RMSE for the training set are 0.982 and 0.009, and the *R* ^*2*^ and RMSE for the test set are 0.841 and 0.063. The optimal prediction results of RF are shown in Figure 3. The results *Q* ^*2*^ of K-fold cross-validation for training set and test set are 0.886 and 0.823 respectively, where the *K* value is 5.

### 3.3 Results of RBF-SVM

The RBF-SVM method needs to determine the values of some parameters, such as the penalty coefficient *C*, *ε*-insensitive loss function, and the parameter *γ* of the kernel function. As the penalty coefficient used to control the loss function, if *C* is too large, the penalty for false regression predictions is too large, which is easy to lead to overfitting. If *C* is too small, the penalty for false regression predictions is too small, which easily leads to underfitting. As a parameter of the radial basis kernel function, the larger the *γ* is*,* the easier it is to overfit, and the smaller the *γ* is, the easier it is to underfit. The greater the *ε* is, the less the support vector is, and the greater the support vector is.

**FIGURE 4:**
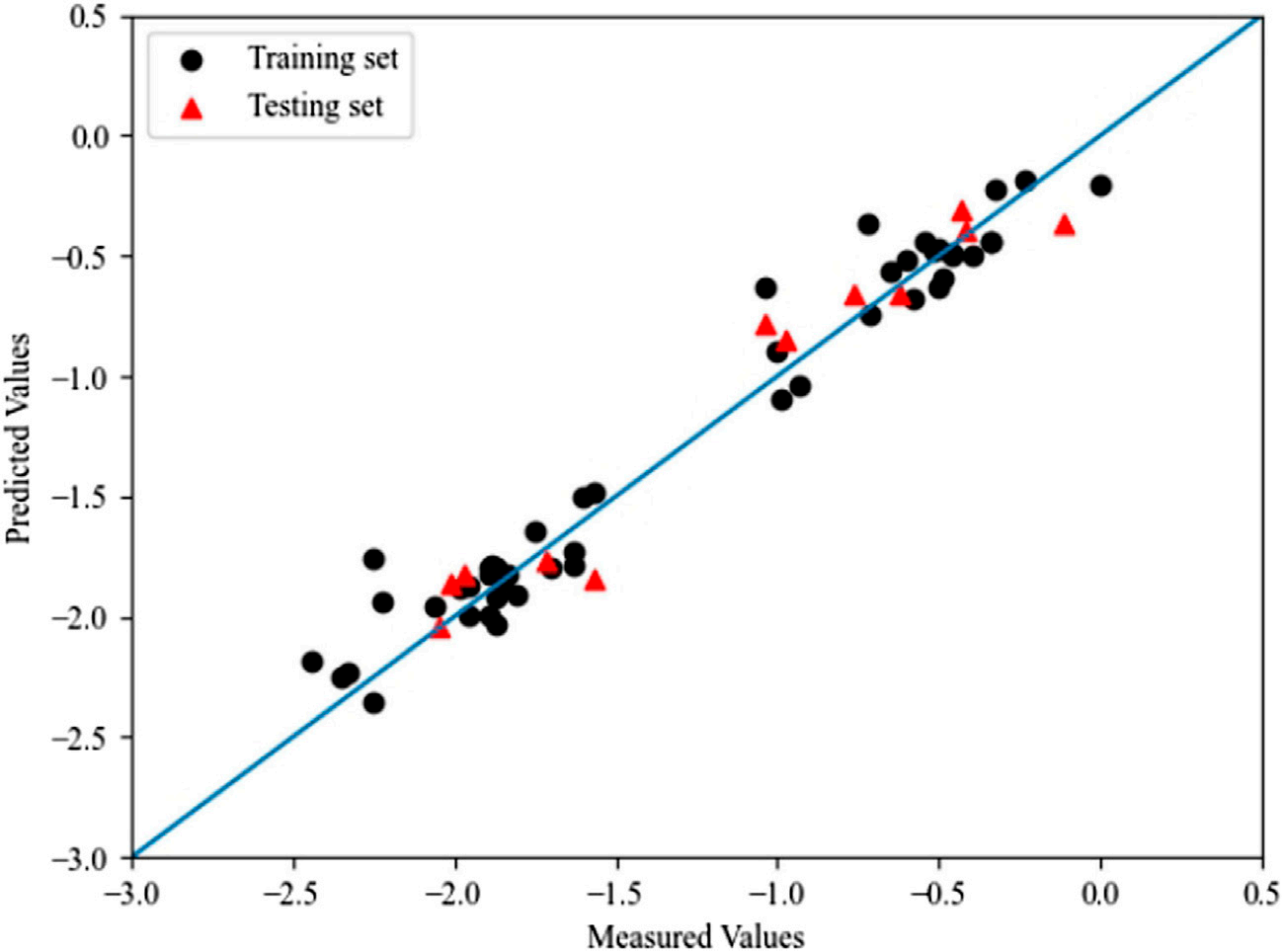
The plot of measured and predicted -lg (IC_50_) by RBF-SVM.

Grid search is the simplest and most widely used hyperparameter search algorithm, which determines the optimal value by looking for all the points within the search range. The optimal values of C and γ were determined by using grid search. The optimal values of *C*, γ and ε are 9.11,10.24 and 0.1, respectively. The optimal prediction results of RBF-SVM are shown in Figure 4. The *R* ^*2*^ and RMSE of the training set and prediction set using RBF-SVM are 0.957,0.022 and 0.944,0.025, respectively. When K-fold (*K* = 5) cross-validation was executed, *Q* ^*2*^ for the training set and the test set are 0.897 and 0.871, respectively.

### 3.4 Results of PSO-SVM

Many parameters need to be determined during the modeling process using RBF-SVM, and PSO algorithm with character of easy implement, high precision, fast convergence can quickly find the optimal solution. Therefore, PSO algorithm was used instead of grid search to find the optimal parameters. The particle swarm size and number of iterations were set to 600 and 1,500, respectively.

**FIGURE 5:**
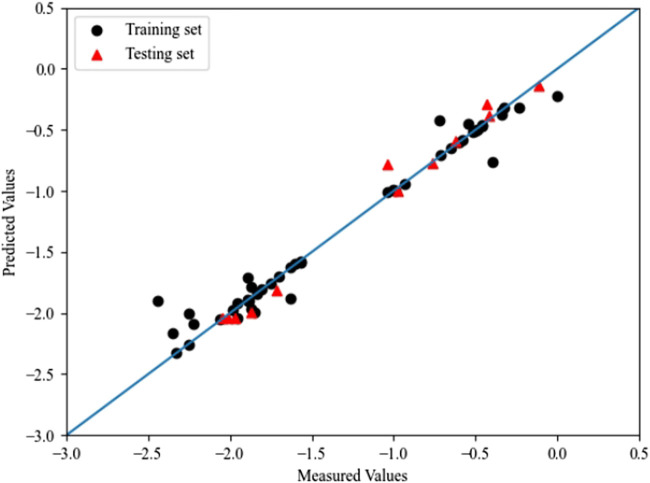
The plot of measured and predicted -lg (IC_50_) by PSO-SVM.

The optimal values of *C*, *γ* and *ε* are 9.13, 1.82 and 0.0, respectively. The *R* ^*2*^ and RMSE of the training and prediction sets using RBF-SVM were 0.966,0.018 and 0.975,0.012, and *Q* ^*2*^ were 0.903 and 0.896, respectively. The optimal prediction results of PSO-SVM are shown in Figure 5.

## 4 Comparison of different results

The prediction results of the four models are shown in Supplementary Table S4, and the K-fold cross-validation results are shown in Supplementary Table S5, where the value of K was set to 5. Moreover, it can be seen from Supplementary Table S4 that the RMSE of PSO-SVM is the smallest, indicating the best degree of fitting.

It can be seen from Supplementary Table S4 that the prediction accuracy and robustness of the nonlinear model established by RF, RBF-SVM and PSO-SVM methods are stronger than that of the linear model HM. However, the prediction accuracy of the model built by RF method on the training set was very high, but the prediction accuracy of the test set was relatively ordinary, which indicates that the model built by RF method is overfitting. The prediction accuracy of the models built by RBF-SVM and PSO-SVM are very high. Among the four models, the model established by PSO-SVM has the strongest prediction ability and stability, for the PSO algorithm can efficiently find more optimized parameters.

## 5 Discussion

In this study, 4 descriptors were selected from 579 descriptors of 60 compounds containing triazole, and 4 QSAR models were established using HM, RF, SVM and PSO-SVM methods to predict the HDAC inhibition. Among the four models, the prediction ability and stability of PSO-SVM are the best, indicating that the model established by PSO-SVM method has a broad application prospect in searching for compounds with significant anti-colon cancer effect, and can be used as an effective method to assist drug design. In addition, this study also revealed four descriptors with significant inhibitory effects on HDAC: Max e-e repulsion for a H-N bond, Max resonance energy for a H-N bond, Max nucleoph react index for a N atom and Min valency of a O atom.

Among the four descriptors, Max e-e repulsion for a h-n bond is the one that has the most significant inhibiting effect on HDAC. Because it is always selected as the top node and has the highest Gini coefficient in the model built by RF. It reflects the repulsion force between electrons and plays a key role in forming the momentum distribution of the final correlated double electrons. The second descriptor is Max resonance energy for an H-N bond. The bond resonance energy represents the contribution of a given bond in a molecule to the topological resonance energy. If a molecule has one or more bonds with a large negative bond resonance energy, the molecule is very chemically reactive. The third descriptor Max nucleoph react index for a N atom is a quantum chemical descriptor, which indicates the strength of covalent bond in the molecule, represents the maximum nuclear reaction index of N atoms. Min valency of a O atom is a quantum chemical descriptor whose scope goes beyond the strength of intramolecular adhesion and accounts for the stability of the molecule and its conformational flexibility. Attempts to increase the valence of the O atom in the substituent may help to reduce the IC50 value of HDAC inhibition.

The compounds numbered 20 and 27 in Supplementary Table S1 have lower IC_50_ values, so other similar compounds with the above descriptors may be novel anti-colon cancer inhibitors that could be designed as potential drugs. Overall, this study revealed four descriptors with significant HDAC inhibition, which could help in the design of novel anti-colon cancer drugs in the future.

## 6 Conclusion

PyCharm Community Edition 2022.2.1 was used for experiments, and the scikit learn library was used to build machine learning models. The models established by RBF-SVM and PSO-SVM have good prediction performance and strong robustness, indicating that the models constructed by RBF-SVM and PSO-SVM have a broad application prospect in the study of the inhibitory effect of triazole-containing compounds on colon cancer. In addition, this study revealed 4 key descriptors that influence the inhibition of HDAC: Max e–e repulsion for a H–N bond, Max resonance energy for a H-N bond, Max nucleoph react index for a N atom and Min valency of a O atom.

In addition to some traditional and common QSAR models, this study also used particle swarm optimization to optimize the SVM model, which greatly improved the prediction accuracy, making the accuracy of the test set increased to 0.975, which will provide guidance and help for the future research on anti-colon cancer drugs.

## Data Availability

The original contributions presented in the study are included in the article/Supplementary Material, further inquiries can be directed to the corresponding author.
