# Supplementary material for: Prediction of histone deacetylase inhibition by triazole compounds based on artificial intelligence
Source: Front Pharmacol. 2023 Nov 15;14:1260349. doi: 10.3389/fphar.2023.1260349 (PMC10684768; doi:10.3389/fphar.2023.1260349)
Supplement: Supplementary file 1 [file Table1.DOCX]

Table 1. Measured and Predicted −lg (IC_50_) of HDAC Inhibition

| 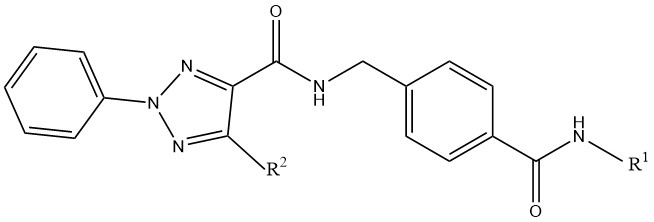 | | | | | | | | | |
| --- | --- | --- | --- | --- | --- | --- | --- | --- | --- |
| Compound | R^1^ | R^2^ | Measured  IC_50_(nM) | Measured  -lg(IC_50_) | Predicted -lg(IC_50_) | | | | |
|  |  |  |  |  | HM | RF | RBF-SVM | PSO-SVM | |
| 1 | OH | - | 3.8 | -0.58 | -0.30 | -0.54 | -0.68 | | -0.58 |
| 2 | Aniline | - | 94 | -1.97 | -1.97 | -1.88 | -1.83 | | -2.08 |
| 3 | OH | C | 4.5 | -0.65 | -0.27 | -0.58 | -0.57 | | -0.65 |
| 4 | Aniline | C | 96 | -1.98 | -1.90 | -1.92 | -1.88 | | -1.98 |
| 5 | OH | NH^2^ | 2.5 | -0.40 | -0.56 | -0.45 | -0.50 | | -0.74 |
| 6 | Aniline | NH^2^ | 43 | -1.63 | -1.89 | -1.76 | -1.78 | | -1.88 |

Table 1. Continued

| 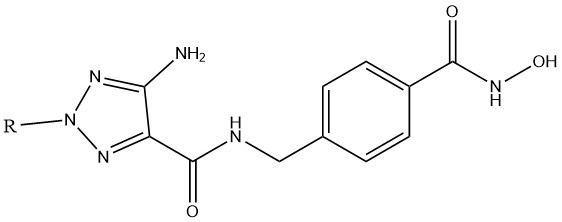 | | | | | | | |
| --- | --- | --- | --- | --- | --- | --- | --- |
| Compound | R | Measured  IC50(nM) | Measured  -lg(IC50) | Predicted -lg(IC50) | | | |
|  |  |  |  | HM | RF | RBF-SVM | PSO-SVM |
| 7 | Pyridine | 11 | -1.04 | -0.59 | -0.78 | -0.63 | -1.01 |
| 8 | 4-fluorophenyl | 5.3 | -0.72 | -0.48 | -0.61 | -0.37 | -0.43 |
| 9 | 3-fluorophenyl | 4.2 | -0.62 | -0.60 | -0.65 | -0.66 | -0.59 |
| 10 | 2-fluorophenyl | 5.2 | -0.71 | -0.69 | -0.64 | -0.75 | -0.71 |
| 11 | 4-chlorophenyl | 2.6 | -0.41 | -0.51 | -0.45 | -0.39 | -0.38 |
| 12 | 3-chlorophenyl | 3.2 | -0.51 | -0.59 | -0.54 | -0.63 | -0.51 |
| 13 | 2-chlorophenyl | 11 | -1.04 | -0.69 | -0.62 | -0.78 | -0.79 |
| 14 | 4-bromophenyl | 2.2 | -0.34 | -0.54 | -0.42 | -0.44 | -0.38 |
| 15 | 4-methoxypheny1 | 2.7 | -0.43 | -0.38 | -0.38 | -0.31 | -0.29 |
| 16 | 3-methoxypheny1 | 3.3 | -0.52 | -0.60 | -0.51 | -0.48 | -0.51 |
| 17 | 4-ethoxyphenyl | 2.2 | -0.34 | -0.40 | -0.37 | -0.44 | -0.35 |
| 18 | 4-(trifluoromethyl)phenyl | 3.1 | -0.49 | -0.65 | -0.57 | -0.59 | -0.49 |
| 19 | 4-isopropylphenyl | 2.9 | -0.46 | -0.49 | -0.48 | -0.49 | -0.47 |
| 20 | 4-ethynylphenyl | 1.7 | -0.23 | -0.46 | -0.28 | -0.18 | -0.30 |
| 21 | 4-(dimethylamino)phenyl | 3.2 | -0.51 | -0.45 | -0.43 | -0.47 | -0.51 |

| 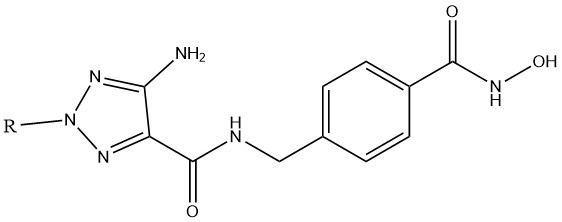 | | | | | | | |
| --- | --- | --- | --- | --- | --- | --- | --- |
| Compound | R | Measured  IC50(nM) | Measured  -lg(IC50) | Predicted -lg(IC50) | | | |
|  |  |  |  | HM | RF | RBF-SVM | PSO-SVM |
| 22 | 4-((dimethylamino)methyl)phenyl | 3.5 | -0.54 | -0.53 | -0.51 | -0.44 | -0.46 |
| 23 | 3,4-dichlorophenyl | 2.9 | -0.46 | -0.54 | -0.52 | -0.49 | -0.46 |
| 24 | 2,3-dichlorophenyl | 9.4 | -0.97 | -0.71 | -0.59 | -0.85 | -1.00 |
| 25 | 3,5-dichlorophenyl | 9.8 | -0.99 | -1.45 | -1.12 | -1.09 | -0.99 |
| 26 | naphthalen-1-yl | 5.8 | -0.76 | -0.29 | -0.40 | -0.66 | -0.78 |
| 27 | naphthalen-2-yl | 1.3 | -0.11 | -0.35 | -0.34 | -0.38 | -0.15 |
| 28 | quinolin-8-yl | 10 | -1.00 | -0.89 | -0.91 | -0.90 | -0.99 |
| 29 | quinolin-6-yl | 1.0 | 0.00 | -0.45 | -0.20 | -0.21 | -0.21 |
| 30 | benzo[d][1,3]dioxol-5-yl | 2.1 | -0.32 | -0.35 | -0.35 | -0.22 | -0.32 |

Table 1. Continued

|  | 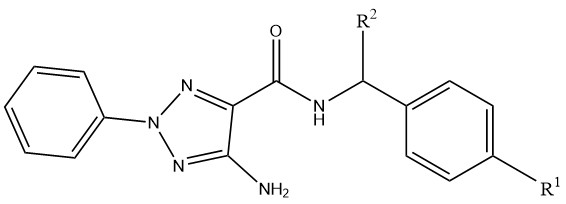 | | | | | | | | |
| --- | --- | --- | --- | --- | --- | --- | --- | --- | --- |
| Compound | | R^1^ | R^2^ | Measured  IC50(nM) | Measured  -lg(IC50) | Predicted -lg(IC50) | | | |
|  |  |  |  |  |  | HM | RF | RBF-SVM | PSO-SVM |
| 31 | | - | 3-(hydroxyamino)-3-oxoprop-1-en-1-yl | 4.0 | -0.60 | -0.56 | -0.55 | -0.52 | -0.61 |
| 32 | | 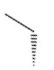 | 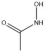 | 40 | -1.60 | -1.33 | -1.39 | -1.50 | -1.59 |
| 33 | | 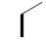 | 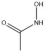 | 8.6 | -0.93 | -1.12 | -0.87 | -1.03 | -0.94 |

Table 1. Continued

Table 1. Continued

| 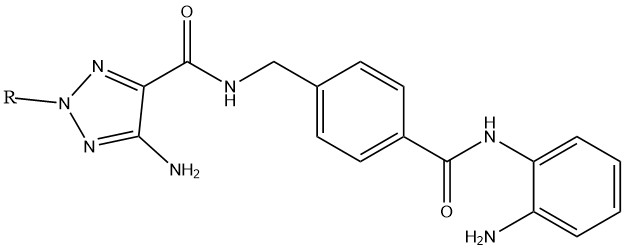 | | | | | | | |
| --- | --- | --- | --- | --- | --- | --- | --- |
| Compound | R | Measured  IC50(nM) | Measured  -lg(IC50) | Predicted -lg(IC50) | | | |
|  |  |  |  | HM | RF | RBF-SVM | PSO-SVM |
| 34 | Pyridine | 74 | -1.86 | -2.02 | -1.91 | -2.03 | -1.97 |
| 35 | 4-fluorophenyl | 77 | -1.88 | -1.95 | -1.89 | -1.79 | -1.90 |
| 36 | 3-fluorophenyl | 90 | -1.95 | -2.01 | -1.94 | -2.00 | -2.04 |
| 37 | 2-fluorophenyl | 71 | -1.85 | -1.97 | -1.87 | -1.85 | -1.99 |
| 38 | 4-chlorophenyl | 102 | -2.01 | -1.98 | -1.70 | -1.86 | -2.04 |
| 39 | 3-chlorophenyl | 115 | -2.06 | -2.03 | -2.07 | -1.96 | -2.05 |
| 40 | 2-chlorophenyl | 52 | -1.71 | -1.88 | -2.05 | -1.77 | -1.81 |
| 41 | 4-bromophenyl | 112 | -2.04 | -2.01 | -2.09 | -2.05 | -2.04 |
| 42 | 4-methoxypheny1 | 74 | -1.86 | -1.85 | -1.86 | -1.92 | -1.79 |
| 43 | 3-methoxypheny1 | 68 | -1.83 | -1.96 | -1.89 | -1.87 | -1.83 |
| 44 | 4-ethoxyphenyl | 78 | -1.89 | -1.84 | -1.92 | -1.99 | -1.90 |
| 45 | 4-(trifluoromethyl)phenyl | 178 | -2.25 | -2.14 | -2.24 | -2.24 | -2.25 |
| 46 | 4-isopropylphenyl | 167 | -2.22 | -1.93 | -2.08 | -2.17 | -2.08 |
| 47 | 4-ethynylphenyl | 78 | -1.89 | -1.97 | -1.87 | -1.80 | -1.71 |
| 48 | 4-(dimethylamino)phenyl | 64 | -1.81 | -1.80 | -1.88 | -1.71 | -1.80 |

Table 1. Continued

| 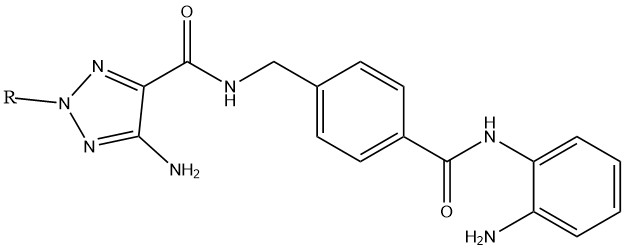 | | | | | | | |
| --- | --- | --- | --- | --- | --- | --- | --- |
| Compound | R | Measured  IC50(nM) | Measured  -lg(IC50) | Predicted -lg(IC50) | | | |
|  |  |  |  | HM | RF | RBF-SVM | PSO-SVM |
| 49 | 4-((dimethylamino)methyl)phenyl | 90 | -1.95 | -1.90 | -1.95 | -1.89 | -1.91 |
| 50 | 3,4-dichlorophenyl | 276 | -2.44 | -2.05 | -2.30 | -2.10 | -1.90 |
| 51 | 2,3-dichlorophenyl | 74 | -1.86 | -1.95 | -1.81 | -1.99 | -2.00 |
| 52 | 3,5-dichlorophenyl | 223 | -2.34 | -2.07 | -2.31 | -2.23 | -2.16 |
| 53 | naphthalen-1-yl | 56 | -1.74 | -1.86 | -1.91 | -1.64 | -1.74 |
| 54 | naphthalen-2-yl | 179 | -2.25 | -1.92 | -2.09 | -1.98 | -2.00 |
| 55 | quinolin-8-yl | 43 | -1.63 | -2.00 | -1.75 | -1.73 | -1.63 |
| 56 | quinolin-6-yl | 37 | -1.56 | -1.97 | -1.91 | -1.84 | -1.57 |
| 57 | benzo[d][1,3]dioxol-5-yl | 50 | -1.70 | -1.95 | -1.79 | -1.79 | -1.71 |

Table 1. Continued

|  | 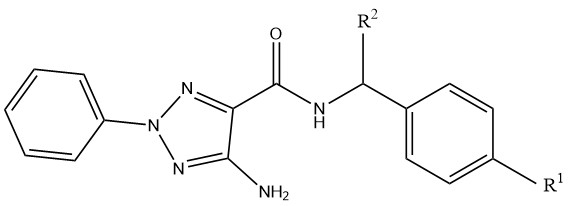 | | | | | | | | |
| --- | --- | --- | --- | --- | --- | --- | --- | --- | --- |
| Compound | | R^1^ | R^2^ | Measured  IC50(nM) | Measured  -lg(IC50) | Predicted -lg(IC50) | | | |
|  |  |  |  |  |  | HM | RF | RBF-SVM | PSO-SVM |
| 58 | | - | 3-(hydroxyamino)-3-oxoprop-1-en-1-yl | 78 | -1.89 | -1.71 | -1.85 | -1.79 | -1.89 |
| 59 | | 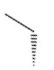 | 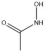 | 212 | -2.32 | -2.14 | -2.19 | -2.23 | -2.32 |
| 60 | | 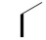 | 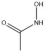 | 37 | -1.56 | -1.62 | -1.64 | -1.48 | -1.57 |
