# Supplementary material for: Prediction of histone deacetylase inhibition by triazole compounds based on artificial intelligence
Source: Front Pharmacol. 2023 Nov 15;14:1260349. doi: 10.3389/fphar.2023.1260349 (PMC10684768; doi:10.3389/fphar.2023.1260349)
Supplement: Supplementary file 2 [file Table2.DOCX]

Table 2. The descriptors selected and their physical-chemical meaning

| Symbol | Physical-chemical meaning |
| --- | --- |
| MERHN | Max e–e repulsion for a H–N bond |
| MREHN | Max resonance energy for a H-N bond |
| MNRIN | Max nucleoph react index for a N atom |
| MVO | Min valency of a O atom |
