# Supplementary material for: Prediction of histone deacetylase inhibition by triazole compounds based on artificial intelligence
Source: Front Pharmacol. 2023 Nov 15;14:1260349. doi: 10.3389/fphar.2023.1260349 (PMC10684768; doi:10.3389/fphar.2023.1260349)
Supplement: Supplementary file 3 [file Table5.DOCX]

Table 5. Comparison of K-fold cross-validation results of different methods.

| Method | Average *Q^2^*  (Training Set) | Average *Q^2^*  (Test Set) |
| --- | --- | --- |
| HM | 0.832 | 0.804 |
| RF | 0.886 | 0.823 |
| RBF-SVM | 0.897 | 0.871 |
| PSO-SVM | 0.903 | 0.896 |
