# Supplementary material for: Prediction of histone deacetylase inhibition by triazole compounds based on artificial intelligence
Source: Front Pharmacol. 2023 Nov 15;14:1260349. doi: 10.3389/fphar.2023.1260349 (PMC10684768; doi:10.3389/fphar.2023.1260349)
Supplement: Supplementary file 4 [file Table3.DOCX]

Table 3. Correlation matrix of the four descriptors

| Descriptor | MERHN | MREHN | MNRIN | MVO |
| --- | --- | --- | --- | --- |
| MERHN | 1 | -0.57 | 0.39 | 0.25 |
| MREHN |  | 1 | -0.63 | -0.05 |
| MNRIN |  |  | 1 | 0.00 |
| MVO |  |  |  | 1 |
