# Supplementary material for: Prediction of histone deacetylase inhibition by triazole compounds based on artificial intelligence
Source: Front Pharmacol. 2023 Nov 15;14:1260349. doi: 10.3389/fphar.2023.1260349 (PMC10684768; doi:10.3389/fphar.2023.1260349)
Supplement: Supplementary file 5 [file Table4.DOCX]

Table 4. Comparison of prediction results of different methods.

| Method | *R^2^* | |  | RMSE | |
| --- | --- | --- | --- | --- | --- |
|  | Training Set | Test Set |  | Training Set | Test Set |
| HM | 0.917 | 0.832 |  | 0.044 | 0.056 |
| RF | 0.981 | 0.856 |  | 0.009 | 0.063 |
| RBF-SVM | 0.957 | 0.944 |  | 0.022 | 0.025 |
| PSO-SVM | 0.966 | 0.975 |  | 0.018 | 0.012 |
